# Probabilistic Genotyping of Single Cell Replicates from Mixtures Involving First-Degree Relatives Prevents the False Inclusions of Non-Donor Relatives

**DOI:** 10.3390/genes13091658

**Published:** 2022-09-15

**Authors:** Kaitlin Huffman, Jack Ballantyne

**Affiliations:** 1 Graduate Program in Chemistry, Department of Chemistry, University of Central Florida, P.O. Box 162366, Orlando, FL 32816-2366, USA; 2 National Center for Forensic Science, P.O. Box 162367, Orlando, FL 32816-2367, USA

**Keywords:** first-degree relatives, mixture deconvolution, probabilistic genotyping, single-cell analysis

## Abstract

Analysis of complex DNA mixtures comprised of related individuals requires a great degree of care due to the increased risk of falsely including non-donor first-degree relatives. Although alternative likelihood ratio (LR) propositions that may aid in the analysis of these difficult cases can be employed, the prior information required for their use is not always known, nor do these alternative propositions always prevent false inclusions. For example, with a father/mother/child mixture, conditioning the mixture on the presence of one of the parents is recommended. However, the definitive presence of the parent(s) is not always known and an assumption of their presence in the mixture may not be objectively justifiable. Additionally, the high level of allele sharing seen with familial mixtures leads to an increased risk of underestimating the number of contributors (NOC) to a mixture. Therefore, fully resolving and identifying each of the individuals present in familial mixtures and excluding related non-donors is an important goal of the mixture deconvolution process and can be of great investigative value. Here, firstly, we further investigated and confirmed the problems encountered with standard bulk analysis of familial mixtures and demonstrated the ability of single cell analysis to fully distinguish first-degree relatives (FDR). Then, separation of each of the individual donors via single cell analysis was carried out by a combination of direct single cell subsampling (DSCS), enhanced DNA typing, and probabilistic genotyping, and applied to three complex familial 4-person mixtures resulting in a probative gain of LR for all donors and an accurate determination of the NOC. Significantly, non-donor first-degree relatives that were falsely included (LRs > 10^2^–10^8^) by a standard bulk sampling and analysis approach were no longer falsely included using DSCS.

## 1. Introduction

The analysis of DNA mixtures containing relatives is increasingly requested in criminal cases [1]. Because of this, many probabilistic genotyping (PG) systems now provide alternative LR propositions in which relatives can be assessed [2]. In these instances (e.g., LR = father vs. hypothetical son), the LRs obtained are significantly decreased due to the common ancestry that must be taken into account (i.e., degree of shared DNA). This differs from the LR algorithms typically reported in forensic DNA analysis where the person-of interest (POI) is evaluated against a random unrelated individual within the population [3]. This is sufficient in a majority of cases, but problems can arise when multiple donors to the same mixture are related or when a non-donor relative of a true donor is being considered as a POI leading to the need for those alternative LR propositions. One option is to utilize a unified LR, which considers both relatives as well as unrelated individuals within the population when calculating the LR [4]. However, because the unified LR assumes > 99.99% of individuals within a population are unrelated, a small impact is often seen on the LRs obtained [5].

The high level of allele sharing seen with familial mixtures can further increase difficulty in assessing the number of contributors (NOC) [6]. This is especially concerning as it is well known that underestimating the NOC to a mixture can result in missed donors. Studies examining simulated complex mixtures with the GlobalFiler^TM^ amplification kit have demonstrated a probability of 86%, 61%, and 17% of 6-person, 5-person, and 4-person mixtures respectively appearing as N−1 or fewer contributors [7]. While peak height was not accounted for in these mixtures, neither was dropout, or stutter masking. Other studies have reported even greater NOC underestimation rates with prepared complex mixtures: 100%, 64%, and 23% of 6-person, 5-person, and 4-person mixtures respectively [8,9]. For mixtures comprised of related individuals, even higher rates underestimating the true NOC are expected.

Previous work on the testing and evaluation of PG systems with complex mixtures which considered the alternative possibility of there being present a relative of one of the donors has demonstrated that non-donor first-degree relatives (FDR) such as a full sibling or parent/child to a true donor can sometimes result in inclusionary LR values [10,11], with some even providing “very strong support” LRs (>10^6^) [11]. A study conducted with STRmix^TM^ examined more complicated familial mixtures comprised of a father, mother, and child, concluding that conditioning the LR on a single parent as well as utilization of the system’s Mx priors function was required to achieve reasonable results [6]. However, this study did not examine the false inclusion of other non-donor relatives, and furthermore, prior information is not always available in actual casework to allow for this conditioning or the user-informed Mx priors function. Other internal crime laboratory validation reports have demonstrated a higher false inclusion rate for relatives compared to unrelated individuals especially with low template samples [12,13,14,15,16,17]. Therefore, fully resolving and identifying each of the individuals present in familial mixtures and excluding related non-donors is an important goal of the mixture deconvolution process and can be of great investigative value. In an attempt to achieve this goal, separation and analysis of each of the individual donors via single cell analysis was carried out by a combination of direct single cell subsampling/enhanced DNA typing [18] and probabilistic genotyping [19] and applied to three complex familial 4-person mixtures resulting in a probative gain of LR for all donors, an accurate identification of the NOC, and elimination of false inclusions of their non-donor relatives.

## 2. Methods

### 2.1. Sample Collection

Buccal swabs were collected from members of three separate families, as well as from two unrelated individuals (U1, U2). Family 1 consisted of a mother (M), father (F), and two children (C1 and C2). Family 2 consisted of a mother (M), father (F), and three children (C1, C2, and C3). Family 3 consisted of 5 full blood siblings (S1, S2, S3, S4, and S5). For each volunteer, a sterile cotton swab was used to swab the inside of the mouth and cheek according to procedures approved by the University of Central Florida’s Institutional Review Board.

### 2.2. Familial Mixture Creation

Equal concentrations of DNA donor extracts from the above familial donors were combined to create desired 2- and 3-person mixtures for later analysis by standard approaches only (i.e., not DSCS). These mixtures consisted of: (i) a father/mother mixture; (ii) a father/child mixture; and (iii) a full sibling/full sibling mixture. Three different 3-person mixtures were also examined including: (i) a father/mother/unrelated individual mixture, (ii) a father/mother/child individual mixture, and (iii) a 3 full-sibling mixture.

Buccal swabs collected from each family were used to create three distinct 4-person mixtures for later use with DSCS as well as standard approaches. Mixture 1 was comprised of the father and mother donors from family 1 as well as 2 unrelated individuals (i.e., F-M-U1-U2). Mixture 2 was comprised of the father, mother, and a child of family 2 as well as 1 unrelated individual (i.e., F-M-C1-U). Mixture 3 was comprised of 4 siblings from family 3 (i.e., S1-S2-S3-S4).

To create each 4-person mixture, the previously collected buccal swabs were agitated in separate aliquots (per donor) of 300 μL TE^−4^ buffer. Each donor solution was then centrifuged at 300 RCF for 7 min to create an epithelial cell pellet. Without disturbing the cell pellet, the supernatant to each solution was discarded and the pellets resuspended with 300 μL of TE^−4^ buffer. The Countess^TM^ II FL (ThermoFisher Scientific, Carlsbad, CA, USA) automated cell counter was then used to determine the cell concentration of each cell suspension. Equal concentrations of the desired donor cell suspensions were combined to create each desired mixture (e.g., mixture 1, mixture 2, or mixture 3). Cell suspensions and mixtures were stored at 4 °C.

### 2.3. Slide Creation

As previously reported, the DSCS approach requires the creation of Gel-Film^®^ microscope slides which the created cell mixtures are deposited on (thus later referred to as mixture slides) as well as a 3M^TM^ adhesive slide which contains adhesive later utilized in the cell collection process [18,19,20,21,22,23]. 

To create the Gel-Film^®^ slides, Gel-Pak^®^ Gel-Film^®^ (WF, ×8 retention level) (Hayward, CA, USA) was attached to clean glass microscope slides by way of the film’s adhesive backing. The film’s clear protective covering was then removed, and 60 μL of a cell suspension mixture (e.g., M-F-U1-U2) was pipetted onto the slide and spread out with a sterile swab. The resulting mixture slide was then stained 1–2 min with Trypan Blue and gently rinsed with nuclease-free water. Mixture slides were allowed to air-dry.

The adhesive slide reservoir was created by attaching 3M^TM^ (Allied Electronics, Fort Worth, TX, USA) adhesive to a clean glass microscope slide by way of double-sided tape. The adhesive backing was removed and the slide stored in a desiccator until needed [18,19,20,21,22,23].

### 2.4. Cell Recovery

A Leica M205C stereomicroscope (190–240× magnification) was used to visualize cells. Cells were collected by way of a sterile tungsten needle which was first utilized to obtain a small ball of 3M^TM^ adhesive that was then used to adhere selected visualized cells from the mixture slides [18,19,20,21,22,23]. The needle with adhesive and cell(s) was then inserted into a sterile 0.2 mL PCR flat-cap tube containing either 5 μL PunchSolution^TM^ (Promega, Madison, WI, USA) or 1 μL casework direct lysis mixture (Promega, Madison, WI, USA) until the 3M^TM^ adhesive was observed to solubilize. Forty 1- and 2-cell subsamples were collected from 4-person mixtures 1 (M-F-U1-U2) and 2 (M-F-C1-U) and forty 1-cell subsamples were collected from the 4-person mixture 3 (S1-S2-S3-S4).

### 2.5. Direct Lysis/Autosomal Short Tandem Repeat (STR) Amplification of Cells

For mixtures 1 and 2, cells were collected directly into 5 μL PunchSolution^TM^ and incubated at 90 °C → 30 min until the lysis solution evaporated. Cells collected from mixture 3 were collected into a lysis mixture comprised of 1 μL casework direct lysis buffer and 0.025 μL 50X diluted 1-thioglycerol. Samples were then incubated at 70 °C → 10 min.

After cell lysis, the subsamples were amplified using the GlobalFiler^TM^ Express amplification kit (ThermoFisher Scientific, Carlsbad, CA, USA) with a reduced reaction volume and increased cycle number. The GlobalFiler^TM^ Express reaction mix was prepared consisting of 2 μL PCR mix, 2 μL primer mix, and 1 μL 5× AmpSolution^TM^ (Promega, Madison, WI, USA). Samples were amplified using a protocol of 95 °C → 1 min; 32 cycles: 94 °C → 3 sec, 60 °C → 30 sec; 60 °C → 8 min; 4 °C → hold. Positive (1 μL of diluted DNA Control 007 (31.25 pg/μL)) and negative amplification controls (0-cell samples and amplification blanks) were included in each amplification batch [19,23].

### 2.6. Donor Reference Samples and Bulk Mixtures

#### 2.6.1. DNA Isolation and Quantitation

DNA extraction was conducted on reference buccal swabs and 60 μL of each mixture cell suspension using the AutoMate *Express*™ Forensic DNA Extraction System (ThermoFisher Scientific, Carlsbad, CA, USA). Each extraction set contained an extraction blank and was quantified with the Quantifiler^®^ Duo DNA Quantification kit (ThermoFisher Scientific, Carlsbad, CA, USA) using the Applied Biosystems’ 7500 real-time PCR instrument (ThermoFisher Scientific, Carlsbad, CA, USA).

#### 2.6.2. Autosomal STR Amplification (Reference Samples and Mixtures)

The GlobalFiler^TM^ (ThermoFisher Scientific, Carlsbad, CA, USA) amplification kit was used to amplify DNA from reference and bulk mixtures samples. One nanogram of input DNA was targeted, and the amplification protocol used was: 95 °C → 1 min; 29 cycles: 94 °C → 10 sec, 59 °C → 90 sec; 60 °C → 10 min; 4 °C → hold. Each amplification contained a positive and negative amplification control.

### 2.7. PCR Product Detection

GlobalFiler^TM^ or GlobalFiler^TM^ Express amplified product (1 μL) was added to 9.5 μL Hi-Di™ formamide (ThermoFisher Scientific, Carlsbad, CA, USA) and 0.5 μL GeneScan^TM^ 600 LIZ^®^ size standard (ThermoFisher Scientific, Carlsbad, CA, USA). Samples were then injected on the Applied Biosystems’ 3500 Genetic Analyzer using POP-4^TM^ polymer and Module J6 (15 s injection, 1.2 kV, 60 °C). GeneMapper v1.6 software (ThermoFisher Scientific, Carlsbad, CA, USA) was used for analysis.

### 2.8. Probabilistic Genotyping (PG)

#### 2.8.1. Standard Bulk Mixture Probabilistic Genotyping

Probabilistic genotyping software STRmix™ v2.8 (Institute of Environmental Science and Research, Auckland, New Zealand) was previously validated for use with standard bulk mixtures and reference samples. Each mixture was examined both by conditioning the LR on a known donor (i.e., $LR=\frac{P_{r}\left(E|\;POI+known\;donor+N-2\;unknown\;individuals\right)}{P_{r}\left(E|\;known\;donor+N-1\;unknown\;individuals\right)}$) and without conditioning (i.e., $LR=\frac{P_{r}\left(E|\;POI+N-1\;unknown\;individuals\right)}{P_{r}\left(E|\;N\;unknown\;individuals\right)}\;$). The FBI Caucasian database was used for all allele frequencies in all mixture experiments [19]. Various number of contributor (NOC) and sub-source LR propositions were examined such as traditional LRs (i.e., evaluating the POI against unrelated individuals in the population), specific relative LRs (i.e., evaluating the POI against a theoretical related individual in the population), and unified LRs (i.e., evaluating the POI against both related and unrelated individuals in the population). Additionally, known FDR non-donors were tested as the POI in the H_1_ or H_p_ proposition to test for advantageous false inclusions.

When the degree of support for the inclusionary proposition based upon the returned LR is expressed as a qualitative verbal statement, the SWGDAM recommendations are followed [24,25]: LR 2–99 (“limited support”), LR 100–9999 (“moderate support”), LR 10,000–999,999 (“strong support”), LR > 1,000,000 (“very strong support”).

The use of unified LR propositions within STRmix^TM^ requires population settings including the relevant population size and the average number of children per family. The U.S. Census data from 2019 was used to estimate the US Caucasian population of 250,446,756 (i.e., 328,238,523*0.763) [26] and 4 was utilized as the average number of children per family [5].

#### 2.8.2. DSCS Probabilistic Genotyping

Previously validated probabilistic genotyping Software STRmix™ v2.8 for single (or few) cell STR analysis [19,23] was used to obtain high resolution single source DNA profiles from 1 or 2 cell subsamples. For the DSCS specificity studies and the complex mixture studies, each 1- or 2-cell single source subsample was run as a single source $LR=\frac{P_{r}\left(E|\;POI\right)}{P_{r}\left(E|\;unknown\;individual\right)}$. For the complex mixtures’ deconvolution, the top 6 subsamples that returned the highest inclusionary LRs (i.e., log (LR) > 1) for a specific donor were used for replicate analysis.

### 2.9. Description of DSCS Method

**Figure 1:**
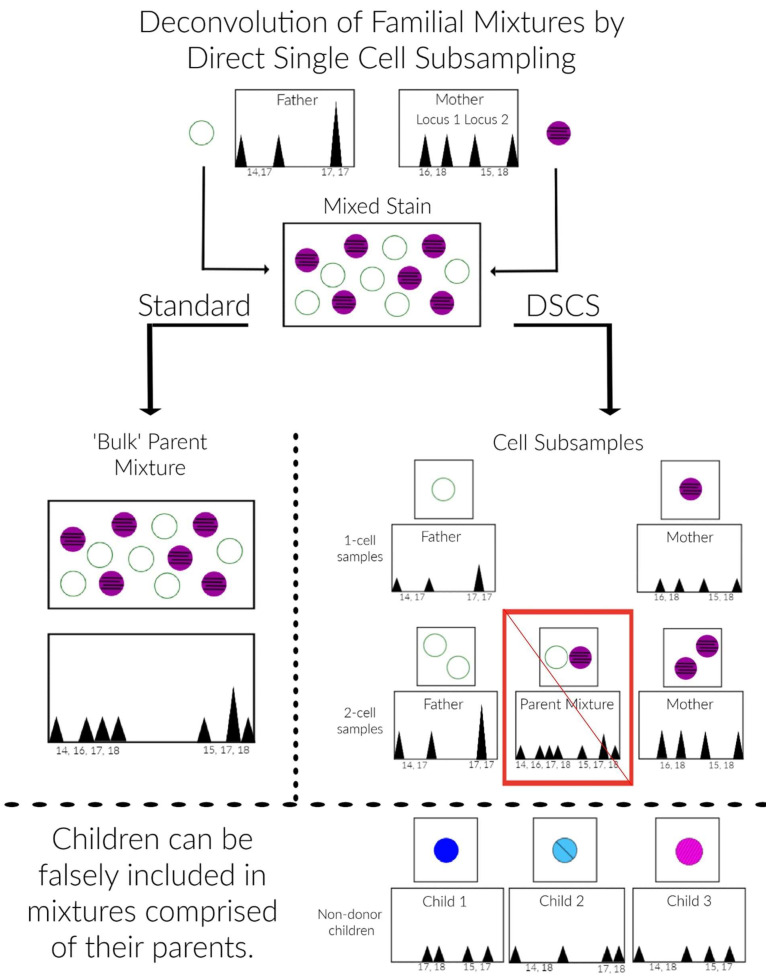
DSCS Analysis Scheme. Standard “bulk” sampling from a mixed stain results in a mixture of DNA from contributors as illustrated with a 1:1 binary DNA mixture of a mother and father’s DNA where each contributor’s genotype cannot be distinguished (**left side**). Without considering peak height, their non-donor children’s genotypes (**bottom**) can be falsely included as contributors to the mixture. Simplified micromanipulation subsampling of the same mixture allows for single- or 2-cell samplings resulting in both single source and mixed DNA profiles of both the mother and father (**right side**). The single source subsamples prevent the false inclusion of non-donor children although the 2-cell mini-mixtures may exhibit the same limitations as standard mixture analysis.

An infographic for the DSCS approach applied, as an example, to a 1:1 2-person father/mother mixture is provided in Figure 1. Standard analysis of the mixed stain results in a mixed DNA profile (Figure 1, left side). Non-donor children of the mother and father can then be falsely included as donors to the mixture (Figure 1, bottom). Direct single cell subsampling of the same mixture allows for collection of 1–2 cell subsamples. This allows for single source profiles of the mother and father to be obtained from the mixture by 1-cell subsamples as well as some 2-cell subsamples. By increasing the number of cells collected in subsampling from 1 to 2, the amount of input DNA doubles thus increasing the probability of achieving a full profile if both cells originate from the same donor. However, some 2-cell subsamples still result in mixed profiles (i.e., 2-cell mini-mixtures) which can pose the same issues as standard familial mixtures (Figure 1, right side). Therefore, it is recommended only single source subsamples be utilized with familial mixtures. The DSCS process referred to for convenience in this study not only encompasses single cell recovery and enhanced DNA typing (Figure 1) but is combined with probabilistic genotyping using STRmix™ software (including the use of its replicate analysis functionality) [19]. 

## 3. Results

### 3.1. False Inclusion of Non-Donor Relatives in 2- and 3-Person Familial Mixtures

Several constitutively different 2- and 3-person first-degree familial DNA mixtures were prepared in the laboratory and tested using standard PG based ‘bulk’ analysis and interpretation methods to confirm the extent to which, and under what circumstances, false inclusions of non-donor relatives could occur, as indicated by the generation of positive log (LR) values. 

For the 2-person mixtures, false inclusions primarily occurred in one of the three mixture types tested (father/mother) for non-donor children when the mother and father were both present within the mixture (Table 1). However, if the mixture was conditioned on there being a single known donor, either the father or mother (which might be possible, for example, in some case scenarios), these false inclusions no longer occurred. 

For the 3-person mixtures, strong false inclusions (i.e., log (LR)s ranging from 5–13) were obtained for all three of the scenarios tested (Table 2). As with the 2-person mixtures, conditioning on one of the known donors helped reduce these false inclusions. However, for the father/mother/child mixture and the 3-sibling mixture, although conditioning decreased the strength of the false inclusions, it also resulted in significantly reducing the strong support for many of the true donors to only limited or moderate support unless the Mx priors function was utilized, in which case strong false inclusions were still seen for non-donor FDRs (Table 3). 

The data from this limited sample set of 2- and 3-person FDR mixtures confirm that such mixtures can result in false inclusions (high LRs) of other first-degree relatives who are not present. Although conditioning on one of the known related donors can sometimes ameliorate this problem it can also reduce the degree of support for some of the other related donors present. 

These initial results provided the impetus to proceed with the direct single cell subsampling (DSCS) approach in an attempt to provide better resolution of such familial mixtures. 

### 3.2. Specificity of DSCS to Distinguish between First-Degree Relatives

**Figure 2:**
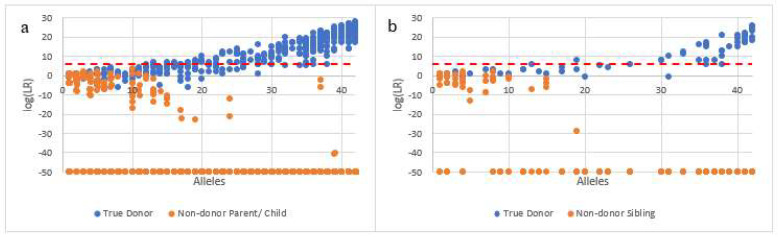
Specificity of DSCS (STRmix™) analysis with first-degree relatives. LR = 0 plotted as −50. Blue circles (known contributor), orange circles (known first-degree relative non-contributor). (**a**) *n* = 455 single source subsamples tested against the false inclusion of the known donor’s parent or child. (**b**) *n* = 70 single source subsamples tested against the false inclusion of the known donor’s sibling. Dashed line indicates the “very strong support” LR threshold of 10^6^ [19].

We first determined if single source cell analysis (1- and 2-cell subsamples), despite the occurrence of allele dropout and other low template DNA artifacts as well as a high degree of allele sharing, could accurately distinguish true donors from their first-degree non-donor relatives (i.e., the LR is calculated as the POI vs. a random unrelated individual). For this, 455 single source cell subsamples (from 7 individuals within 2 separate families) were analyzed with STRmix^TM^ and tested for the inclusion of the true donors versus a parent or child of the true donor (Figure 2a). This was done by substituting the known parent or child non-contributor DNA profiles instead of the known contributors (i.e., substituting the relative for the known in the inclusionary proposition (i.e., H_1_ or H_p_)) and calculating the LR for each of the two situations. Seventy single source subsamples were also tested against the false inclusion of the known donor’s sibling (Figure 2b). Known donor log (LR)s to single source subsamples increased as allele recovery increased and the number of non-contributor false positives with an LR > 1 also decreased as the allele count increased. Non-contributor FDRs did not exceed the “very strong” support LR threshold goal of 10^6^ for unrelated individuals (dashed line) although several cells from non-donor relatives with ≤10 alleles did return LRs ≥ 1 [19]. The majority of these false positives had a log (LR) between 1 and 2 indicating ‘uninformative’ or ‘limited support’ [24,25]. 

The specificity study confirmed that DSCS can distinguish and identify cells originating from FDRs, so long as sufficient alleles (>10 alleles) are detected in the subsamples. 

### 3.3. DSCS Applied to Complex First-Degree Relative Mixtures

Once it was determined that single source subsamples could accurately distinguish known contributors from their first-degree relatives (Figure 2), complex 4-person mixtures were analyzed using both standard approaches and DSCS. The recovered LRs were compared to one another as well as with the maximum recoverable LR (i.e., 1/RMP, the reciprocal of the reference random match probability). The hypothesis was that DSCS should be capable of eliminating (or, at least, reducing) the false inclusion of non-donor relatives to such mixtures. Four-person mixtures containing FDRs were chosen as they were likely to represent some of the most complex mixtures that could be encountered in cases and that we were able to analyze using standard PG approaches as well as by DSCS. These mixtures comprised (1) a father/mother/2 unrelated individuals mixture, (2) a father/mother/child/unrelated individual mixture, and (3) a 4-sibling mixture. In these experiments 40 × 1- and 2-cell subsamples were collected from the mother/father mixtures and 40 × 1-cell subsamples were collected from the sibling mixture.

#### 3.3.1. Mixture 1: Father/Mother/2 Unrelated Individuals

**Figure 3:**
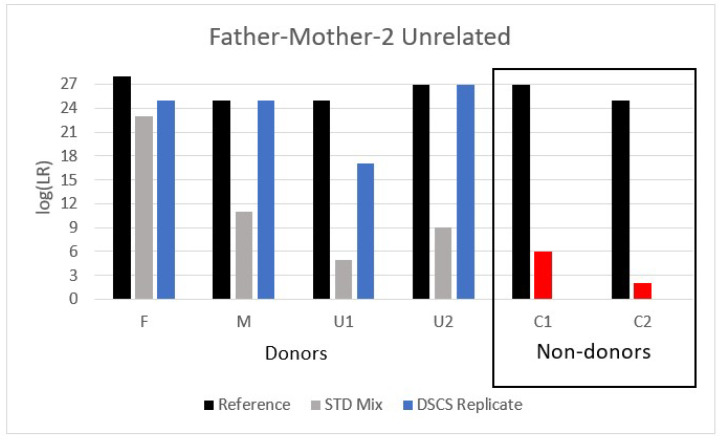
Analysis of a 4-person complex mixture comprised of a father (F), mother (M), and two unrelated individuals (U1, U2) analyzed by DSCS and compared to standard PG mixture analysis (STRmix™). Standard analysis resulted in the false inclusion of known non-donor children (C1, C2) (red shading). The DSCS approach increased contributor log (LR) recovery of known donors and failed to provide a false inclusion of the known non-donor children. For comparison the maximum recoverable log (LR) (i.e., 1/RMP) obtained from reference samples is shown.

The first father + mother containing mixture analyzed (father (F) + mother (M) + 2 unrelated individuals (U1, U2)) using standard bulk analysis resulted in the false inclusion of both of their non-donor children (C1, C2), (assuming an *a priori* accurate assumption of it being a 4-person mixture) though one child’s inclusion (C2) only provided moderate support per SWGDAM’s verbal qualifiers (Figure 3) [24,25]. However, with DSCS, improved genotype recovery as evidenced by increased LRs was obtained for all true donors as well as a finding of no support for the inclusion of either of the non-donor children. 

Notably, if case context permitted the assumption of either the mother or father as a being present in the mixture allowing the LR to be conditioned on the inclusion of one of them, then the non-donor children would no longer be falsely included (Supplementary Table S1). It is also interesting to note that if, as could possibly occur due to overlapping alleles, the mixture was to be misidentified *a priori* as a 3-person mixture (i.e., regarded as an N−1 mixture despite the mixture’s true state being N (i.e., 4) then the 2 unrelated individuals (U1, U2) could be falsely excluded as donors to the mixture, while conditioning the wrongly assumed N−1 mixture on any one of the known donors resulted in no support for any of the other (known) donors in the majority of cases (Supplementary Table S1). 

Since many PG systems now provide alternative LR propositions in which relatives can be assessed, the mixture was then analyzed as $LR=\frac{P_{r}\left(E|\;POI\;+\;3\;unknown\;individuals\right)}{P_{r}\left(E|\;POI’s\;child\;+\;3\;unknown\;individuals\right)}\;$. Therefore, if a parent was the POI (H_p_/H_1_) then H_d_/H_2_ would be a hypothetical child of that parent. For this father/mother/2 unrelated individuals mixture, using alternative LR propositions for relatives gave strong/very strong support for the inclusion of known donors and was uninformative or provided no support for the inclusion of the known non-donors (assuming the correct NOC = 4 was utilized) (Supplementary Table S2). However, if NOC = N−1 (i.e., 3 contributors were assumed) the alternative LR propositions did not improve upon the previous NOC = 3 findings in which the 2 unrelated individuals (U1, U2) would be falsely excluded as donors to the mixture, while conditioning on any one of the known donors resulted in no support for any of the other known donors in the majority of cases (Supplementary Table S2). 

Furthermore, the NOC to the mixture was determined correctly to be four by DSCS since cells from 4 different individual contributors were identified. In contrast, using a commonly used standard approach to determining NOC_min_, (the minimum number of contributors) namely electropherogram inspection for the maximum number of alleles detected at any locus and dividing by two and rounding up, would have determined the NOC_min_ to be three.

#### 3.3.2. Mixture 2: Father/Mother/Child/1 Unrelated Individual

**Figure 4:**
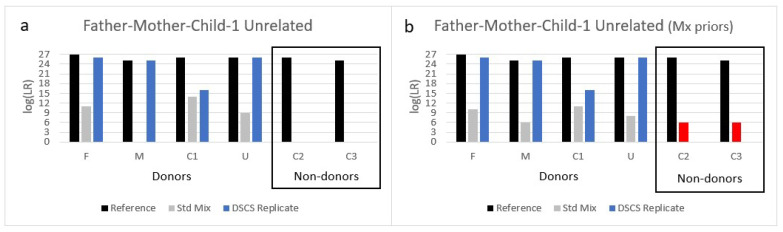
Analysis of a 4-person complex mixture comprised of a father (F), mother (M), their child (C1), and an unrelated individual (U) analyzed by DSCS and compared to standard PG mixture analysis (STRmix™). (**a**) Without any prior information, the mother (M) is falsely excluded as a contributor to the mixture. (**b**) If the M_x_ priors function is utilized, the mother is no longer falsely excluded, but now her known non-donor children (C2, C3) are falsely included (red shade). The DSCS approach increased contributor log (LR) recovery of known donors and prevented the false inclusion of known non-donor children. For comparison the maximum recoverable log (LR) (i.e., 1/RMP) obtained from reference samples is shown.

The second father + mother containing complex mixture analyzed comprised of a father (F) + mother (M) + child (C1) + unrelated individual (U). This mixture, when analyzed by standard bulk analysis without any *a priori* contextual knowledge of the presence of family members in it, resulted in the false exclusion of the mother (Figure 4a, Supplementary Table S3). However, if the Mx priors function is utilized as recommended by STRmix™ instead of the standard method due to unintuitive mixture weights [6], then the mother is no longer falsely excluded. Now, however, her known non-donor children (C2, C3) are falsely included (Figure 4b). Conditioning the standard bulk mixture on any of the known donors still results in the false inclusion of the non-donor children though with widely varying support depending upon the POI and the person conditioned (Supplementary Table S4). If the mixture was misidentified as a 3-person mixture (i.e., N−1), then the mother (M) or child (C1) could be falsely excluded from the mixture depending on the LR scenario (Supplementary Table S3). 

Many of the alternative LR propositions used with mixture #1 would not be appropriate for this particular mixture due to the presence of multiple related individuals as true donors. However, if it was unintentionally employed by the analyst in the absence of appropriate contextual information the following results would be obtained. The LR would be calculated for the father (without conditioning) as $LR=\frac{P_{r}\left(E|\;Father\;+\;3\;unknown\;individuals\right)}{P_{r}\left(E|\;Father^{’}s\;child\;+\;3\;unknown\;individuals\right)}\;$. The competing hypothesis then would be considering that the mixture contains DNA from the father’s child rather than the father. However, the ground truth of the mixture is that both the father and a child of the father are included in the mixture. Nevertheless, using this approach, (with the recommended Mx priors function), only limited to moderate support was obtained for the mother’s inclusion in most scenarios while she was falsely excluded if the mixture was conditioned on her known donor child. The known non-donor children were excluded or included with limited/moderate support (data not shown). Given that the aforementioned scenario is an inaccurate representation of the true state of the mixture, the calculation of a unified LR would be more relevant as it accounts for relatives as well as unrelated individuals in relation to the POI. When a unified LR approach was utilized, however, minimal insignificant differences were seen when compared to the sub-source LRs obtained without accounting for relatives (Supplementary Tables S5 and S6 compared to Supplementary Tables S3 and S4). 

Notwithstanding the above different standard approaches to interpreting this bulk familial mixture, the DSCS approach, once again, improved genotype information recovery for all true donors (i.e., increased LRs) while no support was obtained for false inclusion of the known related non-donors (Figure 4). Furthermore, the minimum NOC (NOC_min_) to the mixture was once again determined correctly to be four by DSCS since cells of 4 different individual contributors were identified. 

#### 3.3.3. Mixture 3: Sibling 1/Sibling 2/Sibling 3/Sibling 4

**Figure 5:**
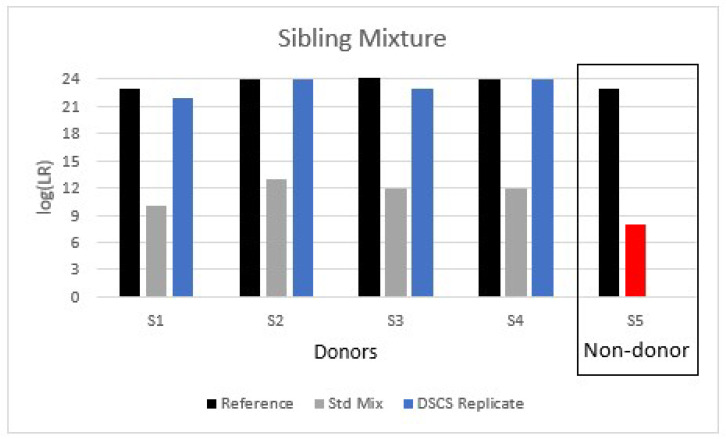
Analysis of a 4-person complex mixture comprised of four full-siblings analyzed by DSCS and compared to standard PG mixture analysis (STRmix™). Standard analysis resulted in the false inclusion of a known non-donor sibling (S5) (red shading). The DSCS approach increased contributor log (LR) recovery of known donors and prevented the false inclusion of the known non-donor sibling. The maximum recoverable log (LR) (i.e., 1/RMP) obtained from reference samples is shown.

The final mixture analyzed in this study was a sibling mixture that comprised four full siblings, S1 + S2 + S3 + S4. This mixture resulted in the false inclusion of a 5th non-donor sibling (S5) when analyzed as a 4-person mixture with standard approaches (Figure 5). Moreover, due to the very high level of allele sharing, the mixture appeared to be that of a 2-person mixture if peak heights were not considered, and potentially a 3-person mixture if peak heights were considered. Therefore, the mixture was analyzed according to multiple NOC propositions (N, N−1, N−2: 4, 3 and 2 respectively) (Supplementary Table S7). With conditioning (N and N−1), the non-donor sibling (S5) was still falsely included with anywhere from limited to strong support depending upon the LR scenario. In one instance, conditioning (on S3) resulted in only limited to moderate support for 2 true donors as well. If analyzed as N−2 contributors (i.e., 2) conditioning resulted in no support for the presence of any of the known donors. 

In terms of adjustments made to the standard analysis to take into account potential relatives, using an alternative LR propositions approach such that the LR is calculated as $LR=\frac{P_{r}\left(E|\;POI\;+\;3\;unknown\;individuals\right)}{P_{r}\left(E|\;POI’s\;sibling\;+\;3\;unknown\;individuals\right)}\;$ would, like mixture #2, again be an inappropriate calculation for the mixture composition (since it is entirely comprised of relatives). Therefore, a unified LR was applied instead which resulted in minimal insignificant differences when compared to the sub-source LRs obtained without accounting for relatives (Supplementary Table S8). 

When DSCS was applied to the sibling mixture, full or near full DNA profiles were obtained for all true donors while no profile was obtained for the non-donor sibling (Figure 5). Additionally, by DSCS all four known donors were identified within the mixture whereas standard approaches indicated an NOC_min_ of 2 or 3.

### 3.4. Effect of Conditioning the LR on a Known Donor in First-Degree Relative Mixtures

**Figure 6:**
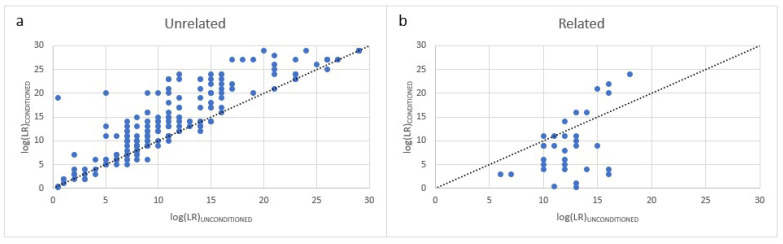
Impact of conditioning the LR on a known donor. (**a**) Mixture comprised of unrelated individuals. (**b**) Mixture comprised of relatives.

Typically, with mixture analysis, conditioning an LR on a known donor improves the strength of inclusion for other true donors as indicated by an increased log (LR) value. This is illustrated in Figure 6a in which 30 mixtures comprised of unrelated individuals were examined during previous validation of the STRmix^TM^ software. Each mixture was analyzed according to the true NOC both with conditioning (e.g., for a 4-person mixture $LR=\frac{P_{r}\left(E|\;POI\;+\;known\;donor+N-2\;unknown\;individuals\right)}{P_{r}\left(E|\;known\;donor\;+\;N-1\;unknown\;individuals\right)}$) and without conditioning (e.g., $LR=\frac{P_{r}\left(E|\;known\;donor\;+\;N-1\;unknown\;individuals\right)}{P_{r}\left(E|\;N\;unknown\;individuals\right)}$). However, when a complex mixture contains 2 or more FDRs, conditioning the LR on one of those relatives often decreases the strength of inclusion for the other relative. Figure 6b illustrates this phenomenon where 7 familial mixtures were examined by conditioning the true donor POI’s LR on their known donor relative’s inclusion as well as without conditioning. This conditioning-dependent reduction in LRs with familial mixtures is likely due to the high level of allele sharing which can result in disproportionately high LRs for the relatives prior to conditioning. This decrease in the LR obtained for an individual when conditioning is employed may provide an indication that related individuals are present within the mixture.

## 4. Discussion and Conclusions

This small study of complex DNA mixtures in which two or more of the donors comprise FDRs confirms the need for DNA analysts to exercise caution when interpreting such mixtures. Most criminal cases will involve calculating an LR for the POI under an assumption that the exclusionary proposition (H_2_ or H_d_) comprises individuals unrelated to the POI. However, as demonstrated in the current work, interpretation becomes more complicated if FDRs of the true donor POI are present in the mixture itself and another FDR, but who is not one of the mixture donors, becomes an alternative POI. There will arise casework situations where the analyst is blind to the fact that the mixture comprises FDR donors and that the presented suspect (POI) is not a donor but is an FDR of some of the true mixture donors. The analyst will likely process and interpret the mixture using a standard H_2_ = unrelated individuals interpretation scheme. We show here that such an approach with 2–4 person familial mixtures containing either the mother + father or multiple siblings can result in the false inclusion of FDRs (i.e., children or other siblings) with some LRs providing very strong support for the inclusionary hypothesis. 

Although this situation of returning false positive LRs for non-donors in some familial mixtures is not ideal there are some potential PG software remedies available to the analyst to help ameliorate it, but these require additional contextual information about the case circumstances and justification for a modified LR calculation. Firstly, if the contextual information indicates that the assumption that there is a known donor present is objectively justifiable then then the mixture can be interpreted by conditioning it on the presence of that known donor. This effectively reduces the complexity of the mixture, especially if the known donor perchance is an FDR of the alternative POI (who is a true non-donor to the mixture), thereby further constraining the possible genotype combinations from the other donors. Although conditioning on the assumption that one of the known related donors was present can sometimes ameliorate this problem with 2–3 person familial mixtures, it can also reduce the degree of support for some of the other related donors present (Table 2). Notably, of the three complex 4-person familial mixtures tested, two of them, despite conditioning, still returned false positive results for the FDR non-donors. Secondly, in addition to conditioning to reduce mixture complexity and constrain the genotype possibilities for other donors, different propositions were used to calculate a variety of different LRs for the 4-person mixtures including specific relative LRs (i.e., evaluating the POI against a related individual in the population), unified LRs (i.e., evaluating the POI against both related and unrelated individuals in the population), and the Mx priors function. None of these resulted in solving the problem of false inclusions of non-donor FDRs for all of the complex 4-person mixtures studied. 

As expected, the high level of allele sharing seen with the type of complex familial mixtures studied here leads to an increased risk of underestimating the number of contributors (NOC) to a mixture. Indeed the 4-person complex mixtures studied could easily be misidentified as 3-person mixtures using the common method of electropherogram inspection for the maximum number of alleles detected at any locus. Interpreting all of these mixtures as 3-person mixtures by standard PG interpretation methods resulted in the false exclusion of true donors, including both FDRs and unrelated individuals. 

All of the above affirms that fully resolving and identifying each of the individuals present in familial mixtures and excluding related non-donors should be an important goal of the mixture deconvolution process. Although the goal is not always readily attainable, attempts should be made to ensure as much as possible that incorrect inclusion inferences are prevented or at least the strength of these false inclusions, as measured by LRs, is minimized. In order to try and achieve that goal, in the present work, instead of analyzing and interpreting complex familial mixtures via the standard bulk analysis approach, separation of each of the individual donors via single cell analysis was carried out by the DSCS process which, in this work, consisted of a combination of direct single cell subsampling, enhanced DNA typing and probabilistic genotyping. Once it was determined that single source cell subsamples could accurately distinguish known contributors from their first-degree relatives (Figure 2), complex familial 4-person mixtures were analyzed using both standard bulk approaches and DSCS. We chose 4-person mixtures as they were some of the most complex mixture types that we could envision in a casework scenario (albeit not encountered on a routine basis) and that we could still analyze using standard PG approaches using the version of STRmix^TM^ (v2.8). By individually analyzing single cells collected from complex familial mixtures, full or near-full single-source DNA profiles were obtained for all true donors resulting in a probative gain of LR information for all donors, thus definitively implicating them as contributors to a mixture while the non-donor relatives were no longer falsely included. As the subsamples collected were single source, there was less risk in obtaining LRs that were disproportionately high or low as could be seen with complex familial mixtures using standard DNA mixture approaches. Furthermore, as familial mixtures have a high degree of allele sharing leading to an underrepresentation of the true NOC, single cell analysis could, and did here, provide an additional way of estimating the NOC. In the present work, the correct NOC was obtained for all three mixtures (i.e., an NOC = 4) based on the number of different DNA profiles recovered by DSCS. Indeed, it is envisioned that the DSCS process *per se,* upon the future development of statistical clustering methods based upon the number of distinguishable genotype-related clusters recoverable from a mixture, could be used to empirically and directly determine the mixture’s NOC. 

If case context does not identify the mother or father as a known donor, then DSCS could also provide a single source DNA profile for them allowing the standard bulk mixture to be conditioned on their inclusion. Such peeling typically results in an improvement in the LR recovery for a mixture comprising unrelated individuals [27]. However as demonstrated in this study a decrease in the LR obtained for an individual when conditioning is employed may provide an indication that related individuals are present within the mixture.

Finally, the implementation of DSCS into routine casework could be achieved using the methodology described herein since only very basic equipment found in most forensic biology laboratories is required. Nevertheless, more widespread implementation of a single cell subsampling, DNA typing and interpretation strategy for mixture analysis would be facilitated by the automation of the DSCS process instead of the manual cell recovery process described here. For example, a combined microfluidics separation and encapsulated digital-droplet single-cell amplification system [28] designed for STR analysis could result in the complete deconvolution of all mixture components to their single source state, thus potentially recovering the complete genotype information present in the sample. 

## Figures and Tables

**Table 1:** Log (LR)s obtained by standard PG analysis of 2-person familial mixtures. Log (LR)s recovered from each of the known contributors or non-contributors treated as the person of interest (POI) using standard analysis (Std Mix). Separate columns show the log (LR)s when computed by conditioning on the presence of either one of the two known contributors. False inclusion log (LR)s obtained from known non-donors exhibiting “very strong”/”strong” support are shaded in red, and those with “limited”/”moderate” support are in orange. F = father, M = mother, C = child, S = sibling.

Father/Mother Mixture				
Family 1	POI	Std Mix	COND F	COND M
Known Donors	F	5		27
	M	12	24	
Non-donors	C1	5	0	0
	C2	13	0	0
**Father/Child Mixture**				
**Family 2**	**POI**	**Std Mix**	**COND F**	**COND C1**
Known Donors	F	18		24
	C1	16	20	
Non-donors	C2	0.4	0	0
	C3	−8	0	0
**2 Siblings Mixture**				
**Family 3**	**POI**	**Std Mix**	**COND S1**	**COND S4**
Known Donors	S1	15		21
	S4	16	22	
Non-donors	S2	−11	0	0
	S3	1	0	0
	S5	2	0	0

**Table 2:** Log (LR)s obtained by standard PG analysis of 3-person familial mixtures. Log (LR)s recovered from each of the known contributors or non-contributors treated as the person of interest (POI) using standard analysis (Std Mix). Separate columns show the log (LR)s when computed by conditioning on the presence of one of the three known contributors. False inclusion log (LR)s obtained from known non-donors exhibiting “very strong”/”strong” support are shaded in red, and those with “limited”/”moderate” support are in orange. F = father, M = mother, C = child, S = sibling, U = unrelated individual.

Father/Mother/Unrelated Mixture					
Family 1	POI	Std Mix	COND F	COND M	COND U
Known Donors	F	11		18	15
	M	9	15		12
	U	12	15	16	
Non-donors	C1	11	0	0	15
	C2	8	0	0	13
**Father/Mother/Child Mixture**					
**Family 2**	**POI**	**Std Mix**	**COND F**	**COND M**	**COND C1**
Known Donors	F	11		23	0.4
	M	13	22		0.3
	C1	16	4	3	
Non-donors	C2	13	1	1	0.4
	C3	12	1	1	0.3
**3 Siblings Mixture**					
**Family 3**	**POI**	**Std Mix**	**COND S1**	**COND S2**	**COND S4**
Known Donors	S1	13		1	11
	S2	14	4		16
	S4	10	6	11	
Non-donors	S3	9	0.7	0.5	3
	S5	5	0.4	0.3	1

**Table 3:** Log (LR)s obtained by standard PG analysis of 3-person familial mixtures, with Mx priors function. Log (LR)s recovered from each of the known contributors or non-contributors treated as the person of interest (POI) using standard analysis (Std Mix). Separate columns show the log (LR)s when computed by conditioning on the presence of one of the three known contributors. False inclusion log (LR)s obtained from known non-donors exhibiting “very strong”/”strong” support are shaded in red, and those with “limited”/”moderate” support are in orange. F = father, M = mother, C = child, S = sibling, U = unrelated individual.

Father/Mother/Child Mixture					
Family 2	POI	Std Mix	COND F	COND M	COND C1
Known Donors	F	13		18	12
	M	11	16		9
	C1	14	14	14	
Non-donors	C2	11	8	9	9
	C3	11	7	8	11
**3 Siblings Mixture**					
**Family 3**	**POI**	**Std Mix**	**COND S1**	**COND S2**	**COND S4**
Known Donors	S1	11		10	12
	S2	13	11		14
	S4	10	11	12	
Non-donors	S3	9	6	6	5
	S5	6	3	3	4

## Data Availability

Requests for additional underlying data can be made to the corresponding author.

## Supplementary Materials

The following supporting information can be downloaded at: www.mdpi.com/article/10.3390/genes13091658/s1, Table S1. Mixture 1: Father/Mother/2 Unrelated individuals, POI vs. unrelated individuals.; Table S2. Mixture 1: Father/Mother/2 Unrelated individuals, POI vs. relative.; Table S3. Mixture 2: Father/Mother/Child/1 Unrelated individual, POI vs. unrelated individuals; Table S4. Mixture 2: Father/Mother/Child/1 Unrelated individual, with Mx priors function.; Table S5. Mixture 2: Father/Mother/Child/1 Unrelated individual, unified LRs.; Table S6. Mixture 2: Father/Mother/Child/1 Unrelated individual, unified LRs (with Mx priors function).; Table S7. Mixture 3: Sibling 1/ Sibling 2/ Sibling 3/ Sibling 4, POI vs. unrelated individuals.; Table S8. Mixture 3: Sibling 1/ Sibling 2/ Sibling 3/ Sibling 4, unified LRs.

## Abbreviations

DSCS: direct single cell subsampling; FDR: first-degree relative; LR: likelihood ratio; NOC: number of contributors; PG: probabilistic genotyping; POI: person-of-interest.
