# Supplementary material for: Probabilistic Genotyping of Single Cell Replicates from Mixtures Involving First-Degree Relatives Prevents the False Inclusions of Non-Donor Relatives
Source: Genes (Basel). 2022 Sep 15;13(9):1658. doi: 10.3390/genes13091658 (PMC9498535; doi:10.3390/genes13091658)
Supplement: Supplementary file 1 [file genes-13-01658-s001.zip › genes-1897820-supplementary.pdf]

Supplementary Tables

Supplementary Table S1. Mixture 1: Father/Mother/2 Unrelated individuals, POI vs. unrelated individuals. Log (LR)s obtained from standard bulk analysis with and without conditioning on known donors. False inclusions obtained with very strong/ strong support or false exclusion (red); False inclusions with limited/ moderate support (orange). NOC: N= 4, N-1 = 3.

	**vs. unrelated.**	POI	Reference	Std Mix	COND F	COND M	COND U1	COND U2
N	Donors	F	28	23		27	23	24
		M	25	11	15		11	20
		U1	25	5	6	5		11
		U2	27	9	10	16	13	
	Related Non-donors	C1	27	6	-2	-13	5	2
		C2	25	2	-1	-12	0.60	-8
N-1	Donors	F	28	23		0	0	19
		M	25	8	0		0	0
		U1	25	0	0	0		0
		U2	27	-7	-12	0	0	
	Related Non-donors	C1	27	-5	0	0	0	0
		C2	25	0	0	0	0	2

Supplementary Table S2. Mixture 1: Father/Mother/2 Unrelated individuals, POI vs. relative. Log (LR)s obtained with and without conditioning on known donors. False exclusions (red); False inclusions with limited/ moderate support (orange). NOC: N= 4, N-1 = 3.

**vs. relative.**		POI	LR type	Reference	Std Mix	COND F	COND M	COND U1	COND U2
N	Donors	F	vs. child	15	12		15	12	13
		M	vs. child	14	6	8		6	12
		U1	vs. unrelated	25	5	6	5		11
		U2	vs. unrelated	27	9	10	16	13	
	Related Non-donors	C1	vs. parent	15	0.85	-5	-21	-2	-5
		C2	vs. parent	14	-5	-3	-20	-7	-15
N-1	Donors	F	vs. child	15	12		0	0	8
		M	vs. child	14	2	0		0	0
		U1	vs. unrelated	25	0	0	0		0
		U2	vs. unrelated	27	-7	-12	0	0	
	Related Non-donors	C1	vs. parent	15	-5	0	0	0	0
		C2	vs. parent	14	0	0	0	0	2

Supplementary Table S3. Mixture 2: Father/Mother/Child/1 Unrelated individual, POI vs. unrelated individuals. Log (LR)s obtained with and without conditioning on known donors. False exclusions (red); inclusion of true donor but with only limited/ moderate support (orange). NOC: N= 4, N-1 = 3.

	vs. unrelated	POI	Reference	Std Mix	COND F	COND M	COND C1	COND U
N	Donors	F	27	11		15	2	13
		M	25	-4	-5		-4	-4
		C1	26	14	7	6		19
		U	26	9	12	11	14	
	Related Non-donors	C2	26	-5	-5	-2	-4	-4
		C3	25	-4	-5	-3	-4	-5
N-1	Donors	F	27	11		15	0	13
		M	25	0	9		0	0
		C1	26	14	0	0		19
		U	26	10	12	11	14	
	Related Non-donors	C2	26	0	0	0	0	0
		C3	25	0	0	0	0	0

Supplementary Table S4. Mixture 2: Father/Mother/Child/1 Unrelated individual, with Mx priors function. Log (LR)s vs. unrelated obtained using Mx priors function with and without conditioning on known donors. False inclusion with very strong/ strong support or false exclusion (red); False inclusion with limited/ moderate support or inclusion of true donor with only limited/ moderate support (orange). NOC: N= 4.

	(Mx priors) vs. unrelated	POI	Reference	Std Mix	COND F	COND M	COND C1	COND U
N	Donors	F	27	10		12	9	12
		M	25	6	9		3	7
		C1	26	11	11	9		14
		U	26	8	11	10	11	
	Related Non-donors	C2	26	6	2	4	4	8
		C3	25	6	3	3	3	7

Supplementary Table S5. Mixture 2: Father/Mother/Child/1 Unrelated individual, unified LRs. Log (LR)s (unified) obtained with and without conditioning on known donors. False exclusion (red); inclusion of true donor with only limited/ moderate support (orange). NOC: N= 4, N-1 = 3.

UNIFIED		POI	Reference	Std Mix	COND F	COND M	COND C1	COND U
N	Donors	F	17	10		13	1	12
		M	16	-5	-6		-5	-5
		C1	16	13	6	5		15
		U	16	9	12	11	13	
	Related Non-donors	C2	16	-5	-6	-3	-5	-5
		C3	16	-5	-6	-4	-5	-5
N-1	Donors	F	17	10		13	0	12
		M	16	0	5		0	0
		C1	16	13	0	0		15
		U	16	9	12	10	13	
	Related Non-donors	C2	16	0	0	0	0	0
		C3	16	0	0	0	0	0

Supplementary Table S6. Mixture 2: Father/Mother/Child/1 Unrelated individual, unified LRs (with Mx priors function). LRs (unified) obtained using Mx priors function with and without conditioning on known donors. False inclusion with very strong/ strong support; False inclusion with limited/ moderate support or inclusion of true donor with only limited/ moderate support (orange). NOC: N= 4.

UNIFIFED		POI	Reference	Std Mix	COND F	COND M	COND C1	COND U
N	Donors	F	17	9		12	9	12
		M	16	5	8		2	7
		C1	16	11	10	9		13
		U	16	8	10	9	10	
	Related Non-donors	C2	16	5	1	4	4	7
		C3	16	5	3	3	2	7

genes

MDPI

Supplementary Table S7. Mixture 3: Sibling 1/ Sibling 2/ Sibling 3/ Sibling 4, POI vs. unrelated individuals. Log (LR)s obtained with and without conditioning on known donors. False inclusion with very strong/ strong support or false exclusion (red); False inclusion with limited/ moderate support or inclusion of true donor with only limited/ moderate support (orange).

	vs. unrelated	POI	Reference	Std Mix	COND S1	COND S2	COND S3	COND S4
N	Known Donors	S1	23	10		5	4	9
		S2	24	13	9		10	16
		S3	25	12	6	8		4
		S4	24	12	11	14	5	
	Related Non-donor	S5	23	8	3	3	4	2
N-1	Known Donors	S1	23	11		4	2	9
		S2	24	14	8		9	16
		S3	25	12	6	7		4
		S4	24	12	11	14	3	
	Related Non-donor	S5	23	9	3	2	3	2
N-2	Known Donors	S1	23	11		0	0	0
		S2	24	14	0		0	0
		S3	25	13	0	0		0
		S4	24	13	0	0	0	
	Related Non-donor	S5	23	9	0	0	0	0

Supplementary Table S8. 4 Siblings. Mixture 3: Sibling 1/ Sibling 2/ Sibling 3/ Sibling 4, unified LRs. Log (LR)s (unified) obtained with and without conditioning on known donors. False inclusion with very strong/ strong support or false exclusion (red); False inclusion with limited/ moderate support or inclusion of true donor with only limited/ moderate support (orange).

	UNIFIED	POI	Reference	Std Mix	Cond. S1	Cond. S2	Cond. S3	Cond. S4
N	Known Donors	S1	16	10		4	4	8
		S2	16	13	8		10	14
		S3	16	11	6	7		4
		S4	16	11	10	13	4	
	Related Non-donor	S5	16	8	3	3	4	2
N-1	Known Donors	S1	16	10		3	2	8
		S2	16	13	8		9	13
		S3	16	12	6	7		3
		S4	16	12	10	13	2	
	Related Non-donor	S5	16	8	3	2	2	2
N-2	Known Donors	S1	16	10		0	0	0
		S2	16	13	0		0	0
		S3	16	12	0	0		0
		S4	16	12	0	0	0	
	Related Non-donor	S5	16	9	0	0	0	0
